# High-yield recombinant production of the semaglutide main chain P29 intermediate using SNAC-tagged enterokinase-cleavable fusion peptides

**DOI:** 10.1371/journal.pone.0348509

**Published:** 2026-06-26

**Authors:** Qingyu Qi, Ge Gao, Guodong Li, Xianling Bian

**Affiliations:** 1 Department of Medical Laboratory Technology, School of Medicine, Xi’An Pei Hua University, Xi’an, Shaanxi, China; 2 Shaanxi Proandy Biotechnology Development Company, Xi’an, Shaanxi, China; 3 Department of Biochemistry, Infectious Diseases Translational Research Programme, Yong Loo Lin School of Medicine, National University of Singapore, Singapore, Singapore; University of the Pacific, UNITED STATES OF AMERICA

## Abstract

Type 2 diabetes mellitus (T2DM) is a chronic metabolic disorder characterized by insufficient insulin secretion or impaired cellular response to insulin, resulting in increased blood sugar levels and persistent hyperglycemia. The diabetes-related health expenditure worldwide is estimated to have surpassed 1,000 billion USD according to the new data from the International Diabetes Federation. Semaglutide, a long-acting glucagon-like peptide-1 (GLP-1) receptor agonist, has demonstrated high efficacy in glycemic control and body weight loss, is approved for T2DM and obesity treatment. However, up to now, industrial production of semaglutide remains constrained by limited yield and high cost associated with conventional approaches. Therefore, improving production efficiency while reducing manufacturing cost remains a challenge. In this study, we report a new strategy for obtaining semaglutide main chain P29, also known as Arg^34^GLP-1 (9–37), which is a key intermediate precursor in semaglutide synthesis. The method employs a series of semaglutide-derived helical fusion pro-peptides containing the sequence GSHHWHHHSSGDDDDK, which could be cleaved by a 2-step processing via sequence-specific nickel-assisted chemical protein cleavage followed by enterokinase cleavage. Using this strategy, semaglutide main chain P29 was highly expressed and purified to 98% purity, with yields exceeding 5 grams per liter of broth. This process provides improved productivity compared with previously reported strategies. The work establishes an efficient and scalable platform for semaglutide intermediate production and shows potential for large-scale industrial production.

## Introduction

As of 2021, the global number of diabetes patients was estimated to be 537 million according to the International Diabetes Federation 10th edition report [1,2]. In 2022, an estimated 828 million people (aged 18 years and older) were living with diabetes, and there was an increase of 630 million people (554–713) from 1990 to 2022 [1,3,4]. According to the latest update of the International Diabetes Federation (2025) report, the numbers continue to increase. In 2024, 589 million people aged 20–79 years were living with diabetes, and 3.4 million people died because of diabetes [5]. Therefore, this growing trend has led to a significant increase in the demand for abundant and various antidiabetic drugs, including GLP-1 receptor agonists to increase insulin secretion [6,7], sodium-glucose transport protein 2 (SGLT2) inhibitors to block renal glucose reabsorption [8–10], and dipeptidyl peptidase 4 (DPP-4) inhibitors to prevent GLP-1 breakdown [11,12]. The global cost of diabetes is estimated to have exceeded 1,000 billion USD in health expenditure, representing a 338% increase over the past 17 years [5].

GLP-1 receptor agonists constitute a class of efficient anti-diabetes and anti-obesity drugs, such as liraglutide and semaglutide, that enhance insulin secretion and suppress glucagon [13–15]. They can mimic the action of the endogenous incretin hormone GLP-1, which is secreted from intestinal L-cells in response to food intake [16,17]. Active semaglutide, with 31 amino acids, is a GLP-1 receptor agonist (residues 7–37, the first six amino acids are missing) used to treat type 2 diabetes mellitus (T2DM) and obesity [14,18]. It is a long-acting analog of human GLP-1 that enhances glucose-dependent insulin secretion, suppresses glucagon release, delays gastric emptying, and promotes satiety, ultimately improving glycemic control and aiding in weight loss [11,13,14,19–21]. Until 2023, semaglutide was the GLP-1 receptor agonist identified by the consensus report by the American Diabetes Association (ADA) and the European Association for the Study of Diabetes (EASD) as showing very high effectiveness in both lowering blood glucose and promoting weight loss in individuals with T2DM [22,23]. Numerous synthesis strategies have been explored for semaglutide including conventional solid-phase peptide synthesis [24–27], however, scalable manufacturing with high purity and production efficiency of semaglutide remains challenging. In this paper, we report to express semaglutide main chain P29 (Arg^34^GLP-1 (9–37)), a key intermediate precursor used in the synthesis of the drug semaglutide. The new strategy significantly improves production yield compared to previous strategies [14,23–27]. The semaglutide main chain P29 can be modified by fatty acid conjugation or any other modification [24], conjugating two fatty acids (2FAs) to peptide drugs can improve their pharmacokinetics and therapeutic effects [28,29].

In this study, to address the limitations of current semaglutide production strategies, we developed a novel recombinant approach for expressing semaglutide truncate Arg^34^GLP-1 (9–37) through introducing multimeric semaglutide-derived helical fusion pro-peptides and the linker containing the sequence GSHHWHHHSSGDDDDK. In this design, tandem P29 units are separated by a SNAC motif and an enterokinase recognition sequence, enabling sequential downstream processing through Ni^2+^-assisted chemical cleavage followed by enterokinase digestion. It is worth noting that the SNAC-mediated cleavage mechanism provides a non-enzymatic alternative to conventional protease processing and has been applied for scalable purification of recombinant peptides and small proteins [30–32]. This engineered system enables efficient expression and controlled release of semaglutide main chain P29 from a multimeric precursor, providing a practical and potentially scalable route for semaglutide intermediate production. Compared with previously reported precursor expression strategies [24–27,34], this approach is expected to establish an efficient and scalable platform for semaglutide intermediate precursor production and shows potential for large-scale industrial production.

## Materials and methods

### Plasmids, cloning and expression

Genes encoding pro-peptides and semaglutide main chain P29 with 4 repeats were cloned and inserted into the pET28a (+) vector as pro-peptides fused at the N-terminus of semaglutide main chain P29, and the recombinant plasmid pET28a-ProPT-SEMP29 was constructed in *E. coli* BL21(DE3). The bacteria were induced to grow to the optical density OD600 of 0.8 at 37 °C by 0.6 mM isopropyl β-D-1-thiogalactopyranoside (IPTG) for 8 hours, after which the cell pellets were harvested by centrifugation (8000 rpm, 10 min).

### Inclusion solubilization and chemical and proteinase cleavage

For gram-scale production, an expanded fed-batch approach was employed via high-cell-density fermentation. For inclusion body (IB) preparation, cell pellets were lysed by pressure homogenization. The IBs were subsequently washed with buffer (10 mM Tris-HCl, pH 8.0) and centrifuged at 12000 rpm for 30 min.

Afterward, the IBs were solubilized in chemical cleavage buffer containing 6 M guanidine hydrochloride (GuHCl), 1 mM NiCl₂ and 0.1 M acetone oxime (pH 8.6), followed by incubation at 42°C for 40 hours, the method referred to the article [30,33–35]. Afterward, the pH of the mixture was adjusted to 3.5 using 1 M HCl, and it was ultrafiltered using a 500 Da molecular weight cutoff (MWCO) membrane to remove small-molecule reagents (GuHCl and NiCl₂). The solution was then neutralized to pH 5.0 with 1 M NaOH, and the precipitated propeptide was collected by centrifugation (10,000 × g, 15 min, 4°C).

The multimeric semaglutide main chain P29 precursor tetrapeptides were dissolved in protease cleavage buffer containing 10 mM Tris-HCl (pH 8.6) and enterokinase (Mengbai, Tianjin, China), and then incubated at 37 °C for 5 h. Afterward, the solution was neutralized to pH 5.0, and the precipitate was collected by centrifugation (10,000 × g, 15 min, 4 °C). The purity of the protein was analyzed using 15% SDS–PAGE with Coomassie blue staining. The protein was characterized by MALDI mass spectrometry.

### Purification of semaglutide main chain P29

The crude proteins were dissolved in ultrapure water containing 0.05% trifluoroacetic acid (TFA) and then purified by reversed-phase high-performance liquid chromatography (HPLC) (Nucifera-C18H, 250 × 4.6 mm, Hedera Sci. & Tech). A gradient of water/acetonitrile with 0.1% TFA was applied, with the sample eluted at a flow rate of 1 mL/min using a 0 ~ 70% linear acetonitrile gradient over 22 min at 25°C. The fraction of semaglutide main chain P29 was collected and analyzed by 15% SDS–PAGE with Coomassie blue staining. The protein was characterized by MALDI mass spectrometry. The product yield was determined gravimetrically, and the volumetric productivity was calculated as grams of product per liter of broth (g L ⁻ ¹).

### Circular dichroism (CD) measurement

CD measurements were performed on an MOS-500 spectropolarimeter. The sample was dissolved in ultrapure water to a final concentration of 0.1 μg/μL, after which the supernatant was collected after centrifugation at 12,000 rpm for 10 min. The far ultraviolet (far-UV) detection condition parameters were as follows: start wavelength, 190 nm; end wavelength, 260 nm; step size, 1 nm; repeats, 1; acquisition period, 1 s/point; and cuvette width, 0.1 cm.

First, blank correction was performed. Blank control solution (ultrapure water) was added to the cuvette, and the baseline was measured and subtracted from the subsequent sample data. Next, 300 μL of sample was loaded into the cuvette, and the measurements were performed under conditions identical to those of the blank. After the raw CD data were saved, CDNN software was used to process the CD spectra data to calculate the secondary structure [34] composition, including helix (spiral), antiparallel (anti-parallel β-folding), parallel (parallel β-folding), beta-turn (β-corner) and random coil structures.

### Mass spectrometry

MALDI mass spectrometry was performed according to a previous article [34]. In brief, semaglutide main chain P29 was co-crystallized with a UV-absorbing matrix on a metal target plate. Upon laser irradiation, the matrix absorbs energy and facilitates desorption and ionization of the sample molecules into the gas phase with minimal fragmentation. The ionized molecules are then analyzed on the basis of their mass-to-charge ratio (m/z) via the use of a TOF mass analyzer.

## Results

### Design of a multimeric semaglutide main chain P29 fusion precursor

**Fig 1:**
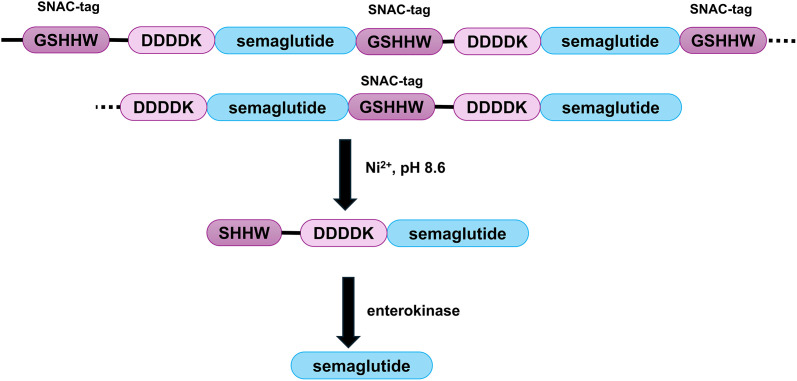
Schematic representation of the two-step purification strategy for recombinant semaglutide. The expression construct comprises tandem repeats of semaglutide main chain peptides 29 (Arg^34^GLP-1 (9-37)) fused with SNAC tags (GSHHW) and enterokinase recognition sites (DDDDK). In the first step, treatment with Ni^2+^ at pH 8.6 induces sequence-specific chemical cleavage at the SNAC tag, partially releasing semaglutide with a remaining DDDDK linker. In the second step, enterokinase specifically cleaves at the DDDDK site, yielding high-purity semaglutide main chain P29. This strategy enables efficient expression and purification of semaglutide in a bacterial system.

To enable high-yield recombinant expression of semaglutide main chain P29 precursor, a multimeric fusion strategy was designed based on tandem repeats of the semaglutide main chain peptide 29 flanked by cleavable linkers and tags (Fig 1). Each semaglutide main chain P29 unit was preceded by an enterokinase recognition sequence (DDDDK) and a N-terminal SNAC tag (GSHHW), allowing precise chemical and enzymatic cleavage. Following expression in *E. coli*, the fusion protein was subjected to a two-step cleavage process. The first step involved chemical cleavage at the SNAC tag sequence using Ni^2+^ under mildly basic conditions (pH 8.6). This selectively removed upstream SNAC-tagged regions, exposing the DDDDK linker. In the second step, enterokinase specifically cleaved at the terminal side of the DDDDK sequence, liberating mature semaglutide intermediate peptides with high sequence fidelity.

### Expression and purification of semaglutide main chain P29

**Fig 2:**
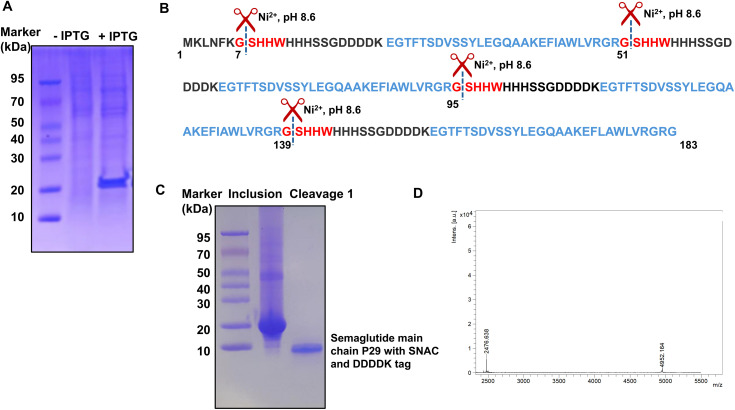
Identification and expression of semaglutide main chain P29 with SNAC and DDDDK tags. (A) SDS–PAGE analysis of recombinant semaglutide main chain P29 fusion protein expression in *E. coli*. Induction with IPTG: + IPTG. (B) Schematic illustration of the designed fusion construct shows multiple tandem repeats of semaglutide sequences flanked by Ni^2+^-cleavable SNAC tags (GSHHW, red) and enterokinase recognition sites (DDDDK). Sites of nickel-assisted cleavage (pH 8.6) are indicated with scissor icons. (C) SDS–PAGE analysis after inclusion body solubilization and Ni^2+^-assisted chemical cleavage (Step 1) shows a successful release of semaglutide main chain P29 with SNAC and DDDDK extensions. (D) MALDI–TOF mass spectrometry shows the identity of the chemically cleaved product.

To verify expression and chemical processing of the semaglutide fusion precursor, sodium dodecyl sulfate polyacrylamide gel electrophoresis (SDS–PAGE) and matrix-assisted laser desorption/ionization–time-of-flight (MALDI–TOF) mass spectrometry were performed. IPTG-induced *E. coli* pellets produced a strong around 20 kDa band corresponding to the multimeric semaglutide main chain P29 fusion protein (Fig 2A). The construct design included four tandem semaglutide repeats separated by SNAC tags (GSHHW) and enterokinase recognition sites (DDDDK), enabling sequential cleavage via Ni^2+^-assisted hydrolysis and proteolysis (Fig 2B). Following inclusion body isolation and nickel-mediated cleavage at pH 8.6, the fusion protein was efficiently processed into shorter fragments. SDS–PAGE analysis of the cleavage product revealed a strong band (Fig 2C), corresponding to semaglutide main chain P29 flanked by residual tags. The identity was confirmed by MALDI–TOF mass spectrometry, which revealed a major peak (Fig 2D), that matched the theoretical mass of the tagged semaglutide intermediate. These results confirm efficient recombinant expression and chemical processing of the multimeric P29 precursor and demonstrate the feasibility of the SNAC-mediated strategy for recombinant semaglutide main chain production.

**Fig 3:**
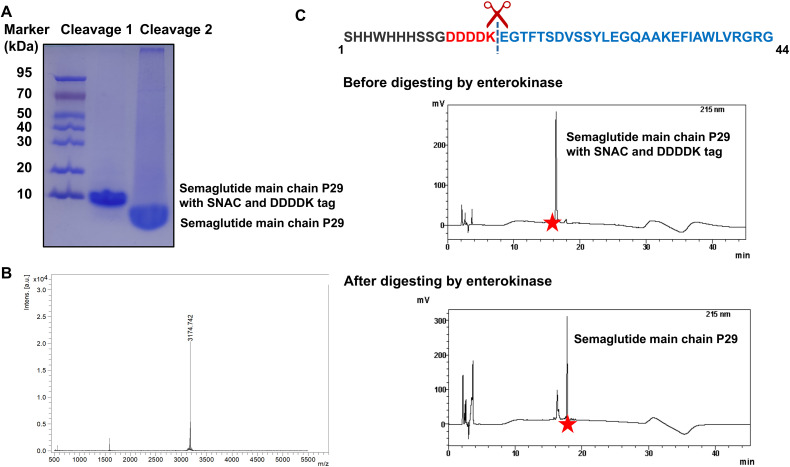
Purification based on enterokinase digestion and identification of semaglutide main chain P29. (A) SDS–PAGE analysis of semaglutide main chain P29 intermediates before and after enterokinase treatment. Lane 1: product of semaglutide main chain P29 with SNAC and DDDDK tags after Ni^2+^-assisted cleavage (Cleavage 1). Lane 2: product of removal of the SNAC and DDDDK extensions after enterokinase digestion (Cleavage 2); semaglutide main chain P29. (B) MALDI–TOF–MS analysis of the purified product after enterokinase digestion. (C) Schematic of the enterokinase cleavage site (red) and corresponding RP-HPLC chromatograms. Top: Before enterokinase digestion, the semaglutide main chain P29 precursor is shown (red star). Bottom: After digestion, a distinct peak shift indicates the release of semaglutide main chain P29 with an altered retention profile (red star).

To obtain native semaglutide main chain P29 from the SNAC- and DDDDK-tagged intermediate, enterokinase digestion was performed following Ni^2+^-assisted cleavage. SDS–PAGE analysis showed a clear decrease in molecular weight, revealing the tag was removed (Fig 3A). MALDI–TOF mass spectrometry further confirmed the identity of the final product (Fig 3B), revealing a major peak matching the theoretical mass of semaglutide main chain P29. Additionally, reverse-phase HPLC revealed a distinct retention time shift after enterokinase cleavage, reflecting the removal of the N-terminal fusion tags (Fig 3C). The purified semaglutide main chain P29 exhibited a single dominant peak and high purity, thus confirming the success of the two-step cleavage strategy. These results validate the efficient enzymatic processing of recombinant semaglutide intermediate and demonstrate a scalable platform for producing GLP-1 precursor using bacterial expression systems. The final semaglutide main chain P29 productivity reached 5.85 g L^-1^, representing improved yield compared with previous reported semaglutide precursor production strategies (Table 1).

**Table 1:** Comparison of strategies for producing the semaglutide main chain precursor.

Methods	Main chain source	Strategies	Typical productivity (g L ⁻ ¹, precursor)	Key features	Representative references
Full solid-phase peptide synthesis (SPPS)	Chemical	Full-length peptide synthesis + lipid conjugation	NA (chemical synthesis)	Very high cost, more steps	[14,23]
Hydrophobic-support-assisted liquid-phase synthesis	Chemical	Alloc-chemistry to the synthesis of Main chain peptide + side chain peptide of semaglutide	NA (chemical synthesis)	High cost, post-synthetic treatment	[24]
Semi-recombinant precursor expression	GLP-1 precursor Lys^26^Arg^34^ GLP1 (11–37)	Solid-phase synthesis + inclusion body expression + fragment condensation coupling	~ 3 g L ⁻ ¹ precursor	Enhance efficiency and yield, refolding and purification of inclusion body	[25]
This work	GLP-1 precursor Arg^34^ GLP1 (9–37)	High-titer fusion precursor + efficient cleavage	~ 5.85 g L ⁻ ¹ precursor	Improved productivity, the SNAC-tag merit	This study

This table summarizes representative chemical and (semi-)recombinant approaches used to obtain the semaglutide main chain peptides. Typical productivity (g L ⁻ ¹) is determined by the gravimetric method; productivity is listed as NA (not applicable) for purely chemical routes; SPPS: solid-phase peptide synthesis; Alloc: allyloxycarbonyl protecting group chemistry; GLP-1: glucagon-like peptide-1. Representative references correspond to the citation numbering in this paper.

### Structural modelling of semaglutide main chain P29

**Fig 4:**
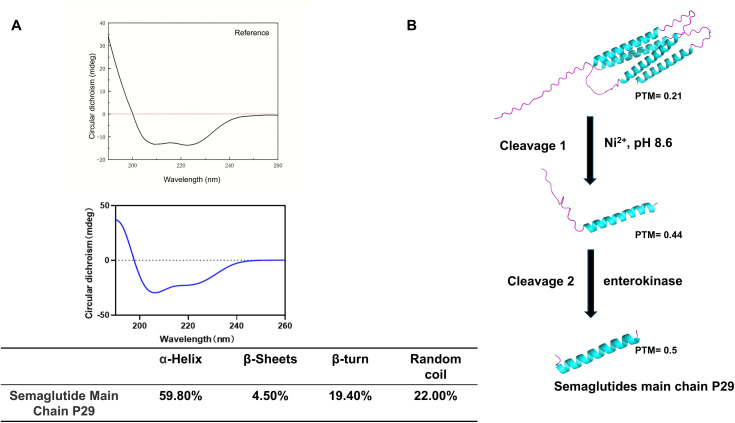
Secondary structure analysis of semaglutide main chain P29. (A) Circular dichroism (CD) spectrums of standard (reference) and purified semaglutide main chain P29, showing a typical α-helical profile with negative ellipticity peaks near 208 nm and 222 nm. Secondary structure analysis revealed a composition of 59.8% α-helices, 4.5% β-sheets, 19.4% β-turns, and 22.0% random coils. (B) Predicted 3D structure of the semaglutide main chain fusion precursor and cleaved product using the AlphaFold server. Cyan: helixs. Purple: Loops. Predicted template model score (pTM) above 0.5 means the overall predicted fold for the complex might be similar to the true structure [36].

To clarify the secondary structure of the recombinantly produced semaglutide main chain P29, circular dichroism (CD) spectroscopy was performed. The CD spectrum exhibited characteristic negative bands at 208 and 222 nm, indicating a predominantly α-helical conformation in the condition, and deconvolution analysis of the spectrum revealed that α-helices comprise approximately 59.8% of semaglutide main chain P29, with minor contributions from β-turns (19.4%), random coils (22.0%), and β-sheets (4.5%). These data suggest a significant α-helical secondary-structure propensity under the tested conditions (Fig 4A). It is well established that short and small peptides in solution generally exist as dynamic conformational ensembles. To explore possible structural trends of the fusion precursor and processed peptide, we use AlphaFold 3 sever to generate computational structural models. The models of the multimeric fusion constructs following simulated two-step cleavage was showed with flexible loops, the isolated P29 segment was predicted to retain α-helical structural features (Fig 4B). Although the predicted template modeling (pTM) score (~0.5) indicates moderate confidence, the computational results are consistent with the α-helical propensity observed by CD analysis (Fig 4B). Taken together, these results suggest that semaglutide main chain P29 exhibits the α-helical secondary-structure propensity under the tested conditions.

## Discussion

The increasing global burden of type 2 diabetes and obesity has created a pressing need for cost-effective production methods of therapeutic peptides such as semaglutide, a long-acting GLP-1 receptor agonist. In this study, we report a new two-step cleavage strategy for obtaining semaglutide main chain P29 (Arg^34^GLP-1 (9–37)), which is a key intermediate precursor in semaglutide synthesis, to form a scalable platform to improve semaglutide yield (Table 1). A multimeric expression construct incorporating SNAC tags (GSHHW) and enterokinase recognition sites (DDDDK) was designed (Fig 1). Using the design, we successfully generated fusion protein precursor in *E.coli*, followed by sequentially obtaining improved yield of tag-free semaglutide main chain P29 intermediate (Figs 2 and 3). And through CD analysis and computer modelling, semaglutide main chain P29 under the tested conditions shows α-helical secondary-structure propensity (Fig 4). The SDS–PAGE analysis confirmed that the designed construct was high-level precursor expressed in *E. coli* as an inclusion body product. MALDI–TOF mass spectrometry revealed mass peaks that closely matched the theoretical values for the intermediate and final products (Figs 2 and 3). RP-HPLC analysis further demonstrated the high purity of semaglutide main chain P29 (Fig 3). Taken together, these results validate the effectiveness of the SNAC/enterokinase-based processing strategy.

Compared with conventional solid-phase peptide synthesis or single-enzyme processing approaches, traditional chemical synthesis of semaglutide predominantly relies on solid-phase peptide synthesis (SPPS), which may generate substantial byproducts and requires extensive purification steps. The recombinant strategy using a short fusion tag design has been reported to yield approximately 3.0 g L ⁻ ¹ of Lys^26^Arg^34^GLP-1 (11–37) via gene recombination (Table 1). Although these recombinant approaches achieved encouraging yields, they rely on refolding of inclusion bodies and column-based purification strategies, which may increase process complexity and dependence on chromatographic resins. In contrast, the present strategy enables direct conversion of inclusion-body fusion protein into a soluble precursor via SNAC-mediated chemical cleavage, followed by removal of chemical reagents through ultrafiltration and subsequent enzymatic digestion to release semaglutide main chain intermediate P29. This approach avoids extensive renaturation procedures and reduces reliance on resin-based purification steps. Furthermore, this SNAC/enterokinase-based strategy provides a scalable and tag-removable approach. The platform offers a practical route to produce the semaglutide core truncate (Arg^34^GLP-1 (9–37)) at gram-per-liter levels (~ 5.85 g L ⁻ ¹ culture). Although lipidation required for full pharmacokinetic activity is not incorporated in the current workflow, the platform establishes a practical foundation for subsequent downstream modifications. In conclusion, this study presents a modular and cost-effective platform for scalable recombinant production of semaglutide main chain P29 intermediate. The approach provides a foundation for downstream modifications such as C-terminal amidation or lipid conjugation and may be extended to other therapeutic peptides requiring precise tag-free recovery from bacterial expression systems.

## Supporting information

S1 File Raw images. (PDF)
