# Supplementary material for: High-yield recombinant production of the semaglutide main chain P29 intermediate using SNAC-tagged enterokinase-cleavable fusion peptides
Source: PLoS One. 2026 Jun 26;21(6):e0348509. doi: 10.1371/journal.pone.0348509 (PMC13308793; doi:10.1371/journal.pone.0348509)
Supplement: S1 File — (PDF) [file pone.0348509.s001.pdf]

High-yield recombinant production of the semaglutide main chain P29 intermediate using SNAC-tagged enterokinase-cleavable fusion peptides

Qingyu Qi et al.

Supporting information

S1 Raw images relevant to Figure 2A, Figure 2C, Figure 3A.

Fig 2A

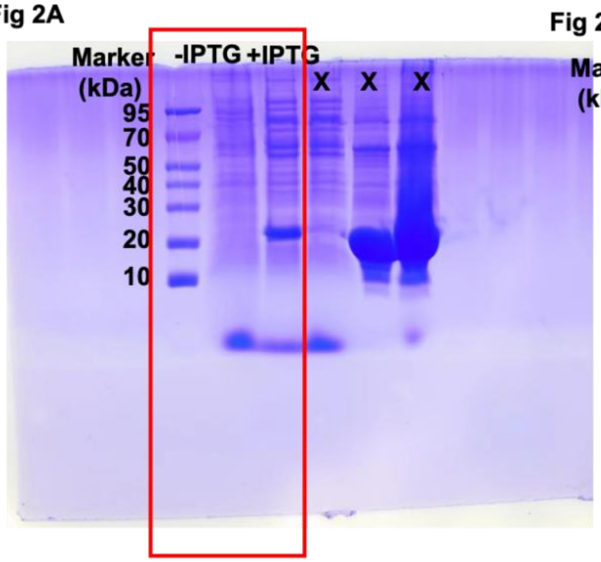

Fig 2C

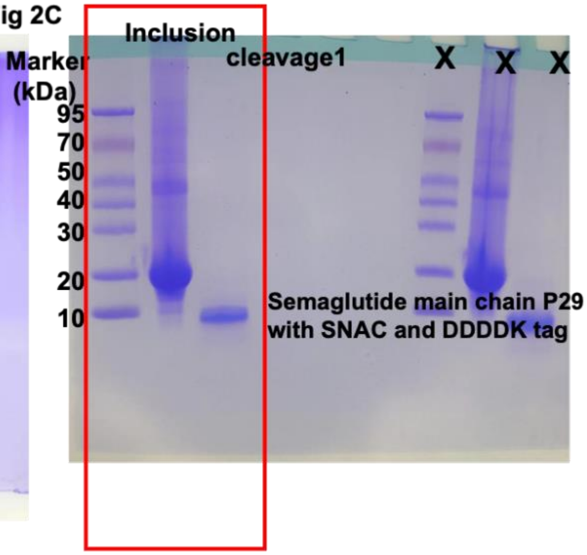

Fig 3A

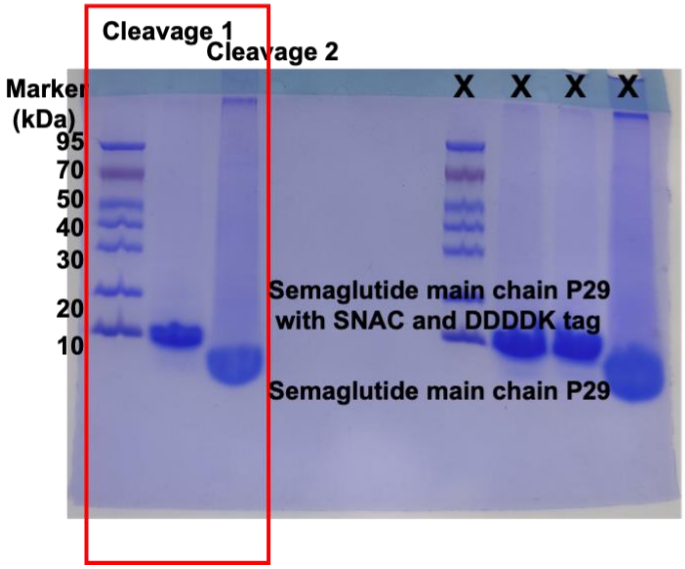

Original uncropped SDS-PAGE gels corresponding to Fig 2A, Fig 2C and Fig 3A. Red boxes indicate the regions used in the main figures. Lanes marked with X contained unrelated samples and were removed during figure assembly.
